# Effects of Methotrexate in a Rabbit Model of In-Stent Neoatherosclerosis: An Optical Coherence Tomography Study

**DOI:** 10.1038/srep33657

**Published:** 2016-09-20

**Authors:** Ruoxi Zhang, Shuyuan Chen, Hui Zhang, Qi Liu, Jianpang Xing, Qi Zhao, Yu Wang, Bo Yu, Jingbo Hou

**Affiliations:** 1 Department of Cardiology, Key Laboratories of Education Ministry for Myocardial Ischemia Mechanism and Treatment, 2nd Affiliated Hospital of Harbin Medical University, Harbin 150086, China

## Abstract

This study used optical coherence tomography (OCT) to investigate the effects of systemic methotrexate, in combination with a drug-eluting stent, on in-stent neoatherosclerosis in a rabbit model. Sirolimus-eluting stents were surgically implanted in the right common carotid arteries of 200 male New Zealand White rabbits; the animals received a high-fat diet, beginning one week before stent implantation. Each animal was randomly assigned to 1 of 4 groups, receiving intravenous injections of either methotrexate (0.4 mg/kg) or placebo weekly for 4 or 12 weeks. Stented arterial segments were harvested after stenting for 4 or 12 weeks, and processed for OCT and histological analysis. Prior to harvesting the arterial segments, blood was collected for the determinations of cytokine levels. Compared with the control animals, the methotrexate-treated animals showed lower rates of lipid-rich intima and per-strut low-signal intensity layers, smaller neointimal areas, and reduced neointimal thickness; larger fibrous cap thicknesses and smaller lumen areas were also seen in the animals receiving methotrexate. The levels of serum interleukin, adhesion molecules, and nuclear factor-κB p65 decreased and IL-10 level increased in the methotrexate-treated animals. Targeting the pro-inflammatory pathways may be an effective way to prevent restenosis without the long-term risk of late thrombosis.

The growing use of stents has improved the results of percutaneous coronary revascularizations in patients^1^,^2^,^3^,^4^,^5^. Furthermore, the use of drug-eluting stents (DESs) has significantly reduced the rates of restenosis and target lesion revascularization compared with the use of bare-metal stents (BMSs). However, the use of DESs is limited by their associated long-term healing delays and increased risks of late stent thrombosis, a catastrophic complication can lead to myocardial infarction or sudden cardiac death^6^,^7^. The development of atherosclerosis, inside stents, which is called “in-stent neoatherosclerosis” (ISNA), has been reported in autopsy^8^, optical coherence tomography (OCT)^9^,^10^,^11^, and angioscopy studies^12^. In recent years, ISNA has been recognized as an important mechanism of late complications, including restenosis and stent thrombosis, in both BMSs and DESs^10^,^11^,^13^.

Growing evidence has shown that atherosclerosis is an inflammatory disease^14^,^15^ and inflammation plays a critical role in neointima formation after coronary artery stenting. The use of immunosuppressants, after BMS deployment, has yielded significant reductions in restenosis, as shown in clinical trials^16^,^17^. Further, previous study has suggested that methotrexate (MTX) can alter cardiovascular risk either directly by influencing atherosclerotic processes via inflammatory responses, or indirectly by influencing cardiovascular risk factors^18^,^19^. However, reports on the relationship between MTX and atherosclerosis are limited^20^,^21^. Therefore, we investigated the anti-restenotic and anti-inflammatory properties of MTX in a rabbit ISNA model using OCT after DES implantation.

## Methods

### Ethics Statement

All animals received humane care, the study protocol was performed following approval of the protocol by the Hospital Scientific Affairs Committee on Animal Research and Ethics (Key laboratories of education ministry for myocardial ischemia mechanism and treatment, 2nd affiliated hospital of Harbin Medical University), and the methods were performed in accordance with the approved guidelines. All rabbits were obtained from the animal center of Harbin Medical University.

### Rabbit Model of ISNA

**Figure 1:**
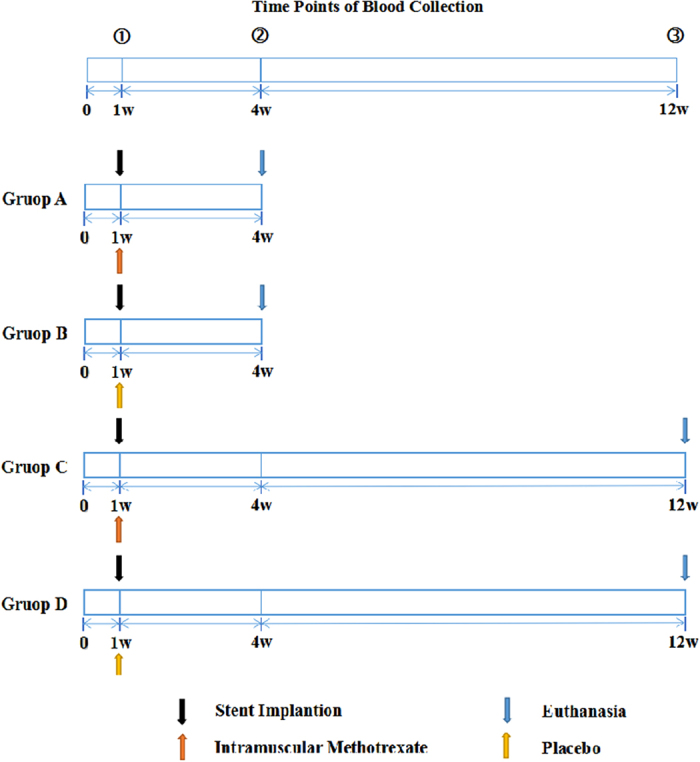
Experimental Study Layout.

The experimental preparation of the atherosclerotic animal model is depicted in Fig. 1. Male New Zealand White rabbits (3–4 kg), 3–4 months of age, were fed an atherogenic diet (1% cholesterol and 6% lard oil, 7.5% yolk powder, Shanghai Capital Bio, Shanghai, China) for 5 or 13 weeks, prior to euthanasia, to induce atherosclerosis. The atherogenic diet was prepared by mixing the cholesterol with rabbit chow in reconstructed pellets that were used as feed. Animals were given free access to water and food. Food intake was recorded daily, and rabbit body weights were determined prior to and at the end of the study. Sirolimus-eluting stents (SESs) were implanted into the right common carotid arteries of the rabbits, 1 week after the induction of the high-cholesterol diet, using a surgical procedure and a coronary stent delivery system, as described previously^22^. OCT was used to scan the right common carotid arteries at 4 or 12 weeks after stent implantation.

### Stent placement and tissue harvest

After surgical exposure of the right carotid artery, rabbits underwent implantation of single SESs (Partner^®^, Lepu Medical, Beijing, China), at 12 atm, in the common carotid artery via the carotid artery. The stents had nominal diameters of 2–2.5 mm, lengths of 8–13 mm, strut thicknesses of 0.18 mm, and an intended stent/artery ratio of 1.2:1. The animals received oral aspirin (40 mg) in combination with oral clopidogrel (75 mg) only for once at 48 h before surgery, followed by only oral aspirin (40 mg) daily until the animal was euthanized; before the interventions, rabbits were also given intravenous heparin (100 IU/kg). At either 4 or 12 weeks after stent implantation, pairs of animals (MTX and placebo) were imaged using OCT to study the different ISNA stages. They were then euthanized and their vessels were processed for histopathology.

### Drug treatment

The animals were randomly allocated to 1 of 4 treatment groups; groups A and C received intravenous injections of MTX (0.4 mg/kg) (Pfizer Pty Limited, Bentley WA, Australia) weekly for 4 or 12 weeks, respectively, and groups B and D received saline injections (for 4 or 12 weeks, respectively). The methotrexate and placebo administrations began on the day of stenting.

### Blood analyses

Rabbits were phlebotomized from ear vein and blood was collected for the determinations of cytokines [interleukin (IL)-6, IL-10, IL-12, monocyte chemotactic protein-1 (MCP-1), and tumor necrosis factor-alpha (TNF-α), intercellular adhesion molecule-1 (ICAM-1), vascular cell adhesion molecule-1 (VCAM-1), and nuclear factor (NF)-κB p65] levels (Fig. 1), prior to stenting and at 4 and/or 12 weeks after stenting. Serum samples were assessed using commercial enzyme-linked immunosorbent assay (ELISA) kits (DuoSet ELISA Development System, R&D Systems, Minneapolis, MN, USA).

### Histology

Histological assessments were carried out using a previously validated methodology^23^. After the follow-up stent imaging, rabbits were euthanized using an overdose of sodium pentobarbital; carotid specimens were excised, fixed in formalin, and then embedded in methyl methacrylate. Four 2-mm sections were obtained from each stent using a tungsten carbide knife. Sections (5-μm thickness) were then cut using an automated microtome and stained with hematoxylin and eosin. Specimens were examined using a DMRAX2 photomicroscope (Leica Microsystems, Milan, Italy), and analyzed using Leica IM 500 image analysis software.

### OCT Imaging

The intracoronary OCT imaging method had been described previously^24^,^25^. Either the time-domain OCT system (M2/M3 System; LightLab Imaging, Westford, MA, USA) or the frequency-domain OCT system (C7-XR OCT Intravascular Imaging System; St. Jude Medical, St. Paul, MN, USA) was used in the present study. All OCT images were stored digitally and submitted to the Key Laboratories of Myocardial Ischemia at the Chinese Ministry of Education (Harbin, China) for off-line analyses.

### OCT image analysis

**Figure 2:**
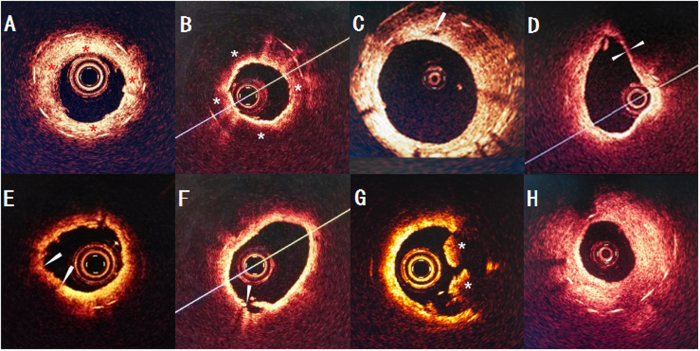
Representative cases with each tissue morphologies assessed with OCT. (**A**) Normal neointima is characterized by homogenous signal-rich band (red asterisk). (**B**) Lipid-laden intima (white asterisk) is observed as a signal-poor band region with poorly delineated border. (**C**) Intraintima neovascularization (white arrow) (**D**) TCFA-like intima (cap thickness, 40 μm) (**E**) OCT-erosion is identified as an irregular lumen surface with attached mural thrombus (arrows) overlying a fibrous plaque. (**F**) OCT image by the disrupted fibrous-cap (white arrow) and a cavity formation inside the plaque. (**G**) OCT image by red Thrombus (white asterisk). (**H**) OCT image shows a severe stenosis.

Assessment of neoatherosclerosis included a determination of the presence of lipids within the stent (Fig. 2), as reported previously^8^,^13^,^26^. A lipid was defined as a diffusely bordered, signal-poor region with rapid signal attenuation. Lipid-laden neointima was defined as neointima containing lipid. Neoatherosclerosis was defined as the presence of lipid-laden neointima inside the stent. Neovascularization was defined as a small vesicular or tubular structure with a diameter ≥50 μm, but ≤300 μm. Thin-cap fibroatheroma (TCFA)–like neointima was defined as lipid-rich neointima having a cap thickness ≤65 μm^10^,^27^.

The OCT analysis included a determination of the presence of lipid-laden intima, the percentage of lipid-rich plaque, and signal attenuation. The findings were compared between animals receiving intravenous MTX injections and those receiving placebo at each period; the differences between the periods were also determined.

OCT images were analyzed by 2 independent investigators who blinded to subject information using proprietary OCT off-line software (Light Lab Imaging, Westford, MA, USA). If there was discordance between the analysts, a consensus reading was obtained from a third independent investigator.

### Correlation between OCT images and histopathology

The correlations between exactly corresponding OCT and histological images were analyzed using the stent edges as anatomical landmarks. The stent struts in each cross-section were numbered and evaluated for the presence of covering tissue. In cases where tissue was found, OCT was used to measure the neointimal hyperplasia thickness. Intra- and interobserver variabilities were determined for the OCT assessments.

### Statistical analysis

The histology and OCT measurement values were expressed as means ± standard deviations. Student’s *t*-test was used to evaluate the similarity of a given measurement between the two groups. The intraclass correlation coefficient was calculated to evaluate the agreement between OCT and histological findings. All analyses were performed using SPSS version 19.0 (SPSS, Chicago, IL, USA); a *P*-value < 0.05 (two-sided) was considered to be significant.

## Results

As shown in Table 1, a total of 157 rabbits with 157 stents were scanned using OCT, and 43 rabbits did not complete the experimental protocol (31 died prematurely from anesthesia accidents, surgical accidents, or serious postoperative infections; 12 developed distal, right carotid artery chronic total occlusions and did not have corresponding OCT images). The remaining 157 rabbits underwent further OCT and histological examinations. Among the groups, no significant differences were seen regarding stent implantation success rates, mean pre-stenting vessel diameters, mean stent diameters, or mean stent lengths.

### OCT findings

The distribution of restenotic tissue structure types and restenotic tissue backscatter types among the four groups are shown in Table 2. The rate of lipid-rich intima occurrence was significantly lower in group C animals than that in group D animals (*P* = 0.041). The restenotic lesions with minimal lumen areas were significantly larger in group A than that in group B animals (*P* < 0.001), and larger in group C than that in group D animals (*P* < 0.001). The restenotic lesions demonstrated significantly less neointimal area in group A than that in group B animals (*P* < 0.001), and in group C than that in group D animals (*P* < 0.001). The neointimal thickness was significantly less in the restenotic lesions in group A than that in group B animals (*P* < 0.001), and in group C than in group D animals (*P* < 0.001).

The OCT analyses of plaque-related neoatherosclerotic findings are shown in Table 3. The plaque fibrous cap thickness was significantly greater in group C than that in group D animals (*P* < 0.001). Further, Table 4 shows the OCT analyses of the non-plaque-related neoatherosclerotic findings. The rate of per-strut, low-signal intensity layers was significantly lower in group C than that in group D animals (*P* = 0.028).

### Agreement between OCT and histopathology images

**Figure 3:**
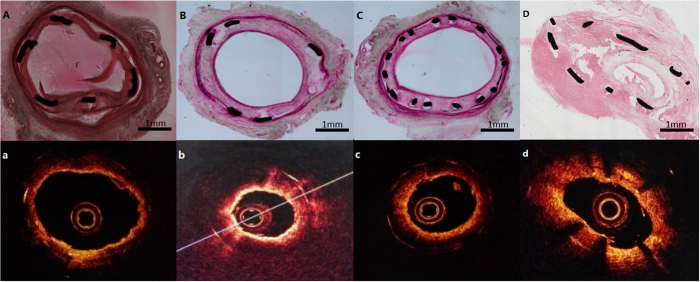
Histological features of ISNA lesions in rabbits and the corresponding OCT images. In-stent neoatherosclerosis: H&E staining (**A–D**) and OCT images (a, b, c, d) of right common carotid arteries at 12 weeks after stenting.

Thirteen representative OCT images and their corresponding histological cross-sections were selected from 13 ISNA lesions to ascertain the agreement between OCT and histopathology findings. Compared with histopathology, OCT measurements of the mean plaque area showed an acceptable correlation, whereas measurements of the fibrous cap thicknesses and lipid arcs, using OCT, were extremely accurate (Table 5 and Fig. 3). The mean plaque thickness and the eccentricity index could not be detected using OCT due to its limited ability to penetrate the lipid content.

### Nuclear transfer of NF-κB, serum inflammatory cytokines and adhesion molecules levels

As shown in Table 6, the levels of pro-inflammatory cytokines (IL-6, IL-12, MCP-1, TNF-α), anti-inflammatory cytokine IL-10, and adhesion molecules (ICAM-1 and VCAM-1) were observed among the four groups at the beginning of the experiment. After 4 weeks of stenting, the serum IL-6, IL-12, MCP-1, TNF-α, ICAM-1 and VCAM-1 levels were significantly lower in group A than those in group B animals, and were also decreased in group C compared with group D animals (all *P* < 0.05). The serum IL-10 level was significantly higher in group A than that in group B animals (*P* = 0.001), and was also increased in group C compared with group D but without significant difference.

**Figure 4:**
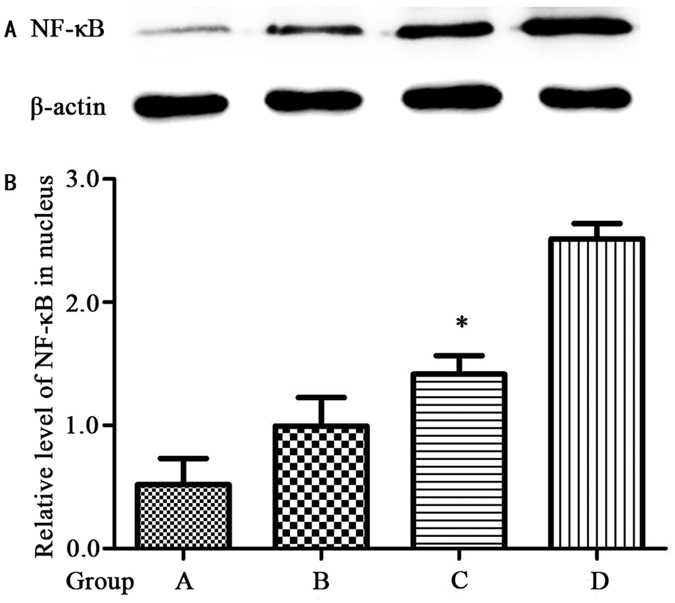
NF-κB p65 Expression in tissue that surrounding the stent. (**A**) Western blot analysis of NF-κB p65 and β-actin. (**B**) Relative level of NF-κB p65 in necleus. *Significance between Group (**C**) and Group (**D**).

At 12 weeks, the serum IL-6, IL-12, MCP-1, TNF-α, ICAM-1 and VCAM-1 levels were significantly lower in group C than those in group D animals (All *P* ≤ 0.001). Again, the serum IL-10 level was remarkably higher in group C than that in group D animals (*P* = 0.038). The nuclear transfer of NF-κB had been tested and shown in Fig. 4. NF-κB p65 expression were significantly lower in group C than that in group D animals at 12 weeks (*P* < 0.05).

## Discussion

To our knowledge, this was the first serial, *in vivo* study using OCT to evaluate the effect of systemic MTX administration in a rabbit model of ISNA. Although the current DESs were effective at reducing the rates of target vessel revascularization, they were coated with anti-proliferative drugs that have undesirable effects, including contributing to persistent fibrin deposition, poor endothelialization, and the induction of inflammatory responses^28^. Moreover, the development of atherosclerotic plaques in stents resulted in more aggressive neointimal responses accompanied with inflammatory reactions, which required long-term dual antiplatelet therapy to reduce the thrombosis risks. Multiple studies had confirmed that the most important risk factor for late stent thrombosis, after DES placement, was withdrawal of antiplatelet therapy^29^.

In previous studies, OCT findings indicated that ISNA were frequently identified in patients with DES in-stent restenosis, especially in those with late in-stent restenosis associated with DESs, including TCFA-containing intima, intimal ruptures, and thrombi^30^. Our observations in DES-treated lesions were similar in this rabbit model. The results from other DES studies had also confirmed that ISNA is a time-dependent pathologic process^9^,^10^,^26^. However, the advent of DESs significantly reduced the rate of post-stenting restenosis, thereby leading to DES implantation becoming the primary mode of coronary revascularization. Regardless, studies had also demonstrated that ISNA occured earlier in DESs than that in BMSs^26^,^31^, especially in restenotic lesions^13^. Recently, an increasing amount of data had supported the importance of ISNA as a potential substrate for late stent thrombi in patients with either BMSs or DESs. As described previously, from the OCT findings in our study, many of the lesions with a layered structure consisted of homogeneous inner layers, with high backscatter, and low-backscatter, attenuated outer layers^32^. Because of the poor endothelialization in DESs, plasma lipoproteins and inflammatory cells easily entered the subendothelial space, and could result in the earlier development of ISNA^8^,^31^. Our results support the concept that inhibition of the inflammatory response may be a promising approach to retard ISNA development.

Earlier studies had reported an association between immunosuppressant use and both neointimal hyperplasia and atherosclerosis^28^,^33^. Inflammatory cells and pro-inflammatory cytokines had been found to play important roles in vascular healing. For example, the number of monocytes adhering to the luminal surface of stented arteries was shown to be linearly correlated with the degree of neointimal hyperplasia^34^. Therefore, the concept of systemic administration of immunosuppressant agents is important. MTX, an immunosuppressant agent, could reduce neointimal formation after coronary stent placement without impeding arterial healing, However, the use of MTX could cause a folic acid deficiency that led to higher homocysteine levels, thereby increasing the risk of cardiovascular disease (CVD)^20^,^35^. On the other hand, Choi and colleagues^21^ reported lower cardiovascular mortality in rheumatoid arthritis patients who treated with MTX, which was ascribed to the anti-inflammatory quality of MTX. The reduction of CVD-related morbidity in MTX-treated patients was in line with the reduced CVD-related mortality in these patients, described by Choi *et al.* which strengthened the hypothesis that reducing inflammation may reduce the risk of CVD. van Halm *et al.* also suggested that MTX treatment was, to a lesser extent, associated with less severe atherosclerosis due to inflammation suppression, which also resulted in a decreased CVD risk^28^. These characteristics have led to enthusiasm for the use of MTX on the prevention of neoatherosclerosis after stenting.

MTX was well-known to suppress several inflammatory pathways by interacting with NF-κB, thus blocking its transcriptional activity^28^,^36^,^37^. NF-κB was a critical signaling molecule during inflammatory process, which facilitated the expressions and secretions of pro-inflammatory cytokines, and then led to a series of inflammatory responses and mucosal damage. It had been identified that, inhibiting the activation of NF-κB could reduce the release of pro-inflammatory cytokines and alleviate the inflammatory response, thereby achieving a therapeutic effect^38^. The reduced transcription of various pro-inflammatory genes resulted in the diminished release of inflammatory cytokines, chemokines, and cell adhesion molecules. Our study confirmed a marked reduction in the nuclear transfer of NF-κB, the release of pro-inflammatory cytokines and the levels of cell adhesion molecules, as well as an increase in the level of anti-inflammatory cytokine IL-10 in stented arteries in animals treated with MTX, compared with placebo-treated animals. We also found that at 4 weeks, IL-10 level was lower in group C and D than that in group A and B. This result can be explained that animals in group C and D received 12-week high-fat diet, which may result in more severe inflammatory response than animals in group A and B. In addition, although IL-10 level was increased in group C compared with group D, no significant difference was found between group C and D, which may be caused by severe inflammatory response and animal individual differences. The MTX dose administered to the rabbits (0.4 mg/kg/week) in this study was approximately equal to the 0.33 mg/kg/week (20 mg for a 60 kg man) of MTX given to humans^39^, which was a relatively high dose in the clinical setting. In this study, no adverse reactions associated with this MTX dose were found in the rabbit model.

### Limitations

The limitations of this study need to be mentioned. First, no animal model can completely mimic human atherosclerosis; thus, careful extrapolation from our results to humans is necessary. Second, although the detection of lipid-rich plaques using OCT has been validated using histopathology studies, data regarding the analysis of neointimal patterns are limited. There is also no absolute consensus among publications regarding the OCT criteria for neoatherosclerosis, and this may affect the reported incidence of this phenomenon. Third, attenuation caused by large amounts of red thrombus obscure the underlying neointimal morphology; OCT-defined TCFA-containing neointima and neointimal ruptures were typically seen proximally or distally (or vice-versa) to the red thrombus and not behind the thrombus, which may lead to an underestimation of the presence of TCFA-containing neointima and neointimal ruptures. Fourth, only one type of stent was tested. Therefore, our results cannot be applied to other types of stents.

### Conclusions

This study demonstrated the feasibility of OCT imaging for quantifying the effects of MTX use in a rabbit model of ISNA. Systemic MTX treatment reduced ISNA formation, compared with placebo, suggesting that the targeting of inflammatory pathways, after stent implantation, may be an effective way to prevent restenosis without the long-term risk of late thrombosis.

## Additional Information

**How to cite this article**: Zhang, R. *et al.* Effects of Methotrexate in a Rabbit Model of In-Stent Neoatherosclerosis: An Optical Coherence Tomography Study. *Sci. Rep.* **6**, 33657; doi: 10.1038/srep33657 (2016).

## Figures and Tables

**Table 1:** Summary of procedural characteristics.

	Group A	Group B	*P*-value	Group C	Group D	*P*-value
	After 4 weeks of stenting		A vs B	After 12 weeks of stenting		C vs D
Number of common carotid arteries treated	50	50	NA	50	50	NA
Number of stents deployed	43	42	NA	43	41	NA
Technical success rate (%)	86	84	1.000	86	82	0.883
Number of deaths	7	8	NA	7	9	NA
Number of CTOs	1	0	NA	5	6	1.000
Mean pre-stenting vessel diameter (mm)	2.53 ± 0.29	2.58 ± 0.35	0.496	2.56 ± 0.33	2.52 ± 0.36	0.6
Mean stent diameter (mm)	2.92 ± 0.43	2.94 ± 0.40	0.832	2.98 ± 0.44	2.99 ± 0.45	0.966
Mean stent length (mm)	15.65 ± 1.60	15.73 ± 1.75	0.828	16.05 ± 1.45	15.69 ± 1.28	0.251
OCT performed	37	41	NA	40	39	NA

N/A, not available; CTO, chronic total occlusion; OCT, optical coherence tomography.

**Table 2:** Optical coherence tomography analyses of the neointima.

	Group An = 37	Group Bn = 41	*P*-value	Group Cn = 40	Group Dn = 39	*P*-value
	After 4 weeks of stenting		A vs B	After 12 weeks of stenting		C vs D
Restenotic tissue structure						
Heterogeneous, n (%)	33 (89.19)	34 (82.93)	0.869	27 (67.50)	18 (46.15)	0.352
Heterogeneous, n (%)	4 (10.81)	7 (17.07)	0.537	13 (32.50)	21 (53.85)	0.304
Layered, n (%)	1 (2.70)	2 (4.88)	0.954	9 (22.50)	20 (51.28)	0.083
Restenotic tissue backscatter						
High, n (%)	31 (83.78)	32 (78.05)	0.867	23 (57.50)	12 (30.77)	0.157
Low, n (%)	6 (16.22)	9 (21.95)	0.779	17 (42.50)	27 (69.23)	0.258
Lipid-rich intima, n (%)	2 (5.40)	4 (9.76)	0.681	10 (25.00)	24 (61.54)	**0.041**
Minimum lumen area, mm^2^	4.59 ± 0.71	3.71 ± 0.52	<**0.001**	3.26 ± 0.59	2.43 ± 0.79	<**0.001**
Stent area, mm^2^	6.03 ± 1.02	5.91 ± 0.61	0.554	6.13 ± 0.61	6.09 ± 0.76	0.797
Neointimal area, mm^2^	1.44 ± 0.61	2.20 ± 0.49	<**0.001**	2.88 ± 0.74	3.66 ± 1.14	<**0.001**
Neointimal thickness, mm	0.21 ± 0.08	0.29 ± 0.08	<**0.001**	0.38 ± 0.10	0.57 ± 0.15	<**0.001**

**Table 3:** Optical coherence tomography analyses of plaque-related neoatherosclerotic findings.

	Group An = 37	Group Bn = 41	*P*-value	Group Cn = 40	Group Dn = 39	*P*-value
	After 4 weeks of stenting		A vs B	After 12 weeks of stenting		C vs D
Total number of plaques	4	9	NA	22	49	NA
TCFA, n (%)	0 (0)	0 (0)	NA	2 (9.10)	9 (18.37)	0.495
LRP, n (%)	2 (50.00)	5 (55.56)	0.99	13 (59.09)	35 (71.43)	0.686
FCT, μm	204.9 ± 62.67	230 ± 41.83	0.054	178.2 ± 54.49	101.4 ± 41.38	<**0.001**
Lipid arc (◦)	98 ± 8.46	110 ± 28.63	0.603	207.77 ± 81.57	233.14 ± 59.89	0.244
Rupture	0 (0)	0 (0)	NA	1 (4.55)	5 (10.20)	0.663
Erosion	0 (0)	0 (0)	2 (9.10)	11 (22.45)	NA	0.331

FCT, fibrous cap thickness; LRP, lipid-rich plaques; TCFA, thin-capped fibroatheroma; N/A, not available.

**Table 4:** Optical coherence tomography analysis of non-plaque-related neoatherosclerotic findings.

	Group An = 37	Group Bn = 41	*P*-value	Group Cn = 40	Group Dn = 39	*P*-value
	After 4 weeks of stenting		A vs B	After 12 weeks of stenting		C vs D
Microvessels, n (%)	0 (0)	1 (2.44)	NA	2 (5.00)	2 (5.13)	0.99
Intimal tears, n (%)	0 (0)	0 (0)	NA	1 (2.50)	5 (12.82)	0.204
Thrombi, n (%)	5 (14.29)	7 (17.07)	0.765	7 (17.50)	11 (28.21)	0.439
Per-strut low-signal intensity layers, n (%)	1 (2.70)	2 (4.88)	0.99	5 (12.50)	17 (43.59)	**0.028**

**Table 5:** Agreement between optical coherence tomography and histology findings.

	OCT (n = 13)	Histology (n = 13)	ICC (95% CI)	*P*-value
Average FCT (mm)	178.52 ± 63.64	107.51 ± 43.16	0.874 (0.639–0.960)	<**0.001**
Average lipid arc (◦)	161.34 ± 73.17	117.75 ± 48.91	0.884 (0.665–0.963)	<**0.001**
LA (mm^2^)	3.45 ± 0.96	1.51 ± 0.39	0.498 (–0.046 to 0.815)	**0.035**

The intraclass correlation coefficient (ICC) was used to evaluate the agreement between the OCT and histology findings.

CI, confidence interval; FCT, fibrous cap thickness; LA, lumen area.

**Table 6:** Serum cytokine profiles at baseline and follow-up.

Cytokine	Group A n = 37	Group B n = 41	*P*-value	Group C n = 40	Group D n = 39	*P*-value
Duration of stenting	After 4 weeks of stenting		A vs B	After 12 weeks of stenting		C vs D
IL-6 (ng/L)						
aseline	94.93 ± 34.66	101.55 ± 32.32	0.385	91.13 ± 32.75	101.75 ± 28.79	0.143
4 weeks	183.62 ± 56.61	426.99 ± 87.83	**0.001**	189.68 ± 56.13	426.22 ± 98.30	**0.001**
12 weeks	—	—	NA	633.63 ± 146.0	1130.19 ± 217.71	**0.001**
IL-10 (μg/L)						
Baseline	8.12 ± 1.71	10.49 ± 11.37	0.213	8.85 ± 2.44	12.26 ± 17.45	0.225
4 weeks	3.73 ± 0.93	3.09 ± 0.75	**0.001**	1.54 ± 0.71	1.28 ± 0.71	0.111
12 weeks	—	—	NA	1.56 ± 0.61	1.25 ± 0.68	**0.038**
IL-12 (μg/L)						
Baseline	1.05 ± 0.32	0.93 ± 0.27	0.078	0.94 ± 0.23	0.91 ± 0.26	0.598
4 weeks	1.24 ± 0.43	1.53 ± 0.50	**0.007**	1.20 ± 0.41	1.54 ± 0.45	**0.001**
12 weeks	—	—	NA	1.72 ± 0.49	1.25 ± 0.68	**0.001**
MCP-1 (μg/L)						
Baseline	21.17 ± 3.04	21.10 ± 3.10	0.970	21.30 ± 2.84	21.67 ± 3.22	0.594
4 weeks	25.79 ± 4.92	28.51 ± 3.32	**0.005**	25.61 ± 3.83	28.79 ± 4.53	**0.001**
12 weeks	—	—	NA	38.30 ± 5.12	53.66 ± 8.16	**0.001**
TNF-α (μg/L)						
Baseline	6.83 ± 0.50	6.77 ± 0.62	0.559	6.84 ± 0.50	6.87 ± 0.59	0.815
4 weeks	7.28 ± 0.44	7.66 ± 0.55	**0.001**	7.20 ± 0.46	7.70 ± 0.44	**0.001**
12 weeks	—	—	NA	8.52 ± 0.65	8.83 ± 0.44	**0.019**
Cell adhesion molecules ICAM-1 (μg/L)						
Baseline	179.32 ± 11.54	178.32 ± 10.06	0.685	177.89 ± 13.05	181.75 ± 8.24	0.126
4 weeks	217.61 ± 27.10	348.22 ± 40.13	<**0.001**	226.26 ± 28.77	356.97 ± 42.21	<**0.001**
12 weeks	—	—	NA	602.26 ± 36.73	757.20 ± 62.60	<**0.001**
VCAM-1 (μg/L)						
Baseline	442.57 ± 41.23	433.49 ± 37.42	0.311	438.48 ± 41.23	451.36 ± 37.39	0.123
4 weeks	505.20 ± 28.21	702.01 ± 40.80	<**0.001**	503.66 ± 38.23	729.68 ± 57.73	<**0.001**
12 weeks	—	—	NA	1636.78 ± 151.24	2034.89 ± 245.99	<**0.001**

IL, interleukin; MCP, monocyte chemotactic protein; TNF, tumor necrosis factor; N/A, not available.
